# High plasma levels of pro-inflammatory factors interleukin-17 and interleukin-23 are associated with poor outcome of cardiac-arrest patients: a single center experience

**DOI:** 10.1186/s12872-020-01451-y

**Published:** 2020-04-15

**Authors:** Yu-Gang Zhuang, Yuan-Zhuo Chen, Shu-Qin Zhou, Hu Peng, Yan-Qing Chen, Dong-Jie Li

**Affiliations:** 1 Department of Emergency Medicine, Shanghai Tenth People’s Hospital, School of Medicine, Tongji University, Shanghai, China; 2 Key Laboratory of Molecular Pharmacology and Drug Evaluation (Yantai University), Ministry of Education, Yantai University, Yantai, China; 3 Department of Pharmacy, Shanghai Tenth People’s Hospital, School of Medicine, Tongji University, 301 Yanchangzhong Road, Jingan District, Shanghai, China

**Keywords:** Post-cardiac arrest syndrome, ROSC, Inflammation, IL-17, IL-23

## Abstract

**Background:**

Systemic inflammation is an important feature of post-cardiac arrest syndrome (PCAS). This study was designed to determine whether the plasma concentrations of some circulating pro-inflammatory cytokines (interleukin-17 [IL-8], IL-22, IL-23 and IL-33) are of value in predicting the outcome of patients after return of spontaneous circulation (ROSC) during the post–cardiac arrest period.

**Methods:**

This was a prospective observational clinical study. In total, 21 patients (survivors, *n* = 10; non-survivors, *n* = 11) who experienced cardiac arrest and successful ROSC with expected survival of at least 7 days were consecutively enrolled from January 2016 to December 2017. Of the 21 enrolled patients, ten survived were designated “survivors”. The other eleven patients died between 2 days and 1 months post ROSC. Venous blood was drawn at three time-points: baseline (< 1 h post ROSC), 2 days post ROSC and 7 days post ROSC. Plasma IL-8, IL-22, IL-23 and IL-33 were determined using commercial enzyme-linked immunosorbent assays.

**Results:**

Plasma creatinine levels, but aspartate aminotransferase (AST) and alanine aminotransferase (ALT) levels, were elevated in non-survivors compared with survivors. Plasma levels of IL-17, IL-22, IL-23 and IL-33 of the 21 total patients did not change at 2 or 7 days post ROSC compared to baseline. In survivors, the plasma levels of IL-17 and IL-23 at 2 or 7 days post ROSC were lower than baseline. In non-survivors, plasma levels of IL-17 increased compared with baseline. Receiver operating characteristic curve analysis showed that the plasma levels of IL-17 and IL-23 at 2 or 7 days post ROSC were able to predict the mortality of PCAS patients, and positively correlated with Acute Physiology and Chronic Health Evaluation (APACHE)-II score and time to ROSC.

**Conclusion:**

These results provide the first evidence that the elevated plasma IL-17 and IL-23 levels are associated with poor outcome in PCAS patients. The role of IL-17/IL-23 axis post ROSC is worth paying attention to in PCAS patients.

**Trial registration:**

Clinicaltrial.govNCT02297776, 2014-11-21.

## Background

Despite the increasing understanding on cardiac arrest and development of resuscitation protocols and cardiopulmonary resuscitation (CPR), admission of cardiac arrest patients to the Intensive Care Unit (ICU) is associated with high morbidity and mortality, and consequently great medical resource consumption [1]. Although it has been previously reported that treating the patients following cardiac arrest with induced hypothermia promoted the resuscitation rate and improved neurological outcome [2], this therapy has been challenged by dozens of recent findings [3]. Moreover, even after successful return of spontaneous circulation (ROSC) from cardiac arrest, the survivors would still face significant morbidity and mortality after hospital discharge [4]. This so-called “post-cardiac arrest syndrome” (PCAS) involves four key components: (1) post-cardiac arrest cerebral ischemia injury, (2) post-cardiac arrest myocardial ischemia and malfunction, (3) systemic ischemia/reperfusion injury, and (4) persistent precipitating pathology [4].

Due to the predicament that critical care interventions of PCAS patient is rather expensive and might be ethically problematic if the outcome is poor, predicting the short- and long-term outcomes in PCAS patients becomes a key challenge in the treatment of PCAS patients. To address this problem, discovering novel biomarkers in PACS patients’ blood is valuable in the clinical therapy of PCAS patients [5]. Donnino et al. reported that effective clearance of blood lactate during the early stage was associated with decreased overall mortality in patients with cardiac arrest [6]. In support of this, survivors from cardiac arrest with good neurological outcome had lower lactate levels in the early stage (< 24 h post ROSC) [7], suggesting that early blood lactate level was an independent predictor of survival and neurological outcomes in PCAS patients. Besides, S-100B, an acidic protein with a calcium binding motif produced by astroglial cells in the brain, is a predictive marker for outcome of patients after cardiac arrest [8]. Neuron specific enolase (NSE), another a neural protein primarily produced by neurons and is leaked into the extracellular space after severe injury, is also a useful predictor of outcome in PCAS patients [9]. Lipocalin, might predict the neurological outcomes of PCAS patients, and its predictive value was equivalent to that of NSE [10].

A study conducted by Bro-Jeppesen et al. showed that systemic inflammation triggered by ischemia and multi-organ failure represents a hallmark of the post-cardiac arrest syndrome (PCAS) in patients with out-of-hospital cardiac arrest [11]. In this study, the authors measured level of interleukin-1β (IL-1β), IL-2, IL-4, IL-5, IL-6, IL-9, IL-10, IL-12, IL-13, tumor necrosis factor-α (TNF-α), interferon-γ (IFN-γ), C-reactive protein, and procalcitonin, and found that levels of IL-6 and IL-10 at baseline were significantly higher in non-survivors compared with survivors [11]. The same group also reported that high IL-6 levels were associated with increased mortality in PCAS patients [12]. Besides, other studies revealed that plasma IL-8 level might be a good marker for predicting the neurological outcome in PCAS patients [8].

In this single-center, case-control study, we measured the plasma levels of IL-17, IL-22, IL-23 and IL-33 in 21 cardiac arrest patients with ROSC at three time-points (baseline [within 1 h post ROSC], 2 days post ROSC and 7 days post ROSC). Although the plasma levels of IL-17, IL-22, IL-23 and IL-33 of the 21 total patients did not change with the course of disease, the plasma levels of IL-17 and IL-23 displayed contrary tendency between the survivors and non-survivors. Moreover, the plasma levels of IL-17 and IL-23 within 48 h post ROSC were potential biomarkers correlating with the neurological injury.

## Materials and methods

### Patients and ethics approval

A total of 21 adult patients (> 18 years-old) were enrolled to the current prospective observational study. These patients were undergone successful cardiopulmonary resuscitation (CPR) and ROSC, and admitted to our intensive care unit within 1 h after CPR/ROSC at the emergency medical center of Shanghai Tenth People’s Hospital, Shanghai from January 2016 to December 2017. Written informed consent was obtained from every patient. The exclusion criteria include: (1) pregnancy; (2) drowning/hanging; (3) neuroendocrine tumor; (3) traumatic brain injury; (4) sepsis; (6) malignant.

The study was reviewed by the local public school research ethics committee and human-investigation committee for the protection of human subjects in Shanghai Tenth People’s Hospital, Tongji University. Moreover, this investigation was registered in *ClinicalTrial.gov* (NCT02297776, 2014-9-21, Link: https://clinicaltrials.gov/ct2/show/NCT02297776?term=02297776&rank=1). This study was conducted in compliance with the “Declaration of Helsinki.” Written informed consents were obtained from all participants.

### Data collection and blood sampling

All the patients with CA and ROSC were treated with intensive medical care. The Acute Physiology and Chronic Health Evaluation (APACHE)-II score was calculated within 1 h of ICU enrolment. Information about time to ROSC, age, gender, past medical history and the cause of CA were collected. Peripheral venous blood was taken from any easily accessible peripheral vein at three time-points post ROSC: 1 h, 2 days and 7 days. Plasma was obtained [13, 14] and frozen at − 80 °C for further experiment [15].

### Kidney and liver function

To monitor the kidney and liver function, the concentrations of creatinine, aspartate aminotransferase (AST) and alanine aminotransferase (ALT) in plasma were determined using an Auto Chemistry Analyzer (Hitachi 7180, Tokyo, Japan) as described previously [16, 17].

### Enzyme-linked immunosorbent assay (ELISA)

Four kinds of pro-inflammatory factors levels in plasma (IL-17, IL-22, IL-23 and IL-33) using commercial (ELISA) kits as described previously [18, 19]. The ELISA kit for IL-17, IL-22 and IL-23 assays were purchased from R&D Systems (catalogue: HS750, D2000 and D3300 respectively, Minneapolis, MN, USA). The IL-23 assay was purchased from ThermoFisher (catalogue: BMS2023–3, Waltham, MA, USA). The optical density (OD) of each reaction was determined at 450 nM using a TECAN Infinite M200 microplate reader (Tecan, Durham, USA) [20–23].

### Statistical analysis

All the results were presented as the mean ± SEM. Categorical variables (e.g., gender and comorbidities) are expressed as percentages. ANOVA test or Mann-Whitney U test was used to test differences among groups [24]. Correlation among data was performed by using the Pearson linear test. Receiver-operating characteristic curves were used to evaluate the pro-inflammatory factors as potential markers of CA outcome. Statistical analyses were conducted with GraphPad Prism software suite (version 5.0, La Jolla, CA, USA) [25]. A *P*-value < 0.05 was considered statistically significant.

## Results

### Plasma pro-inflammatory factors do not change post ROSC in CA patients

**Fig. 1:**
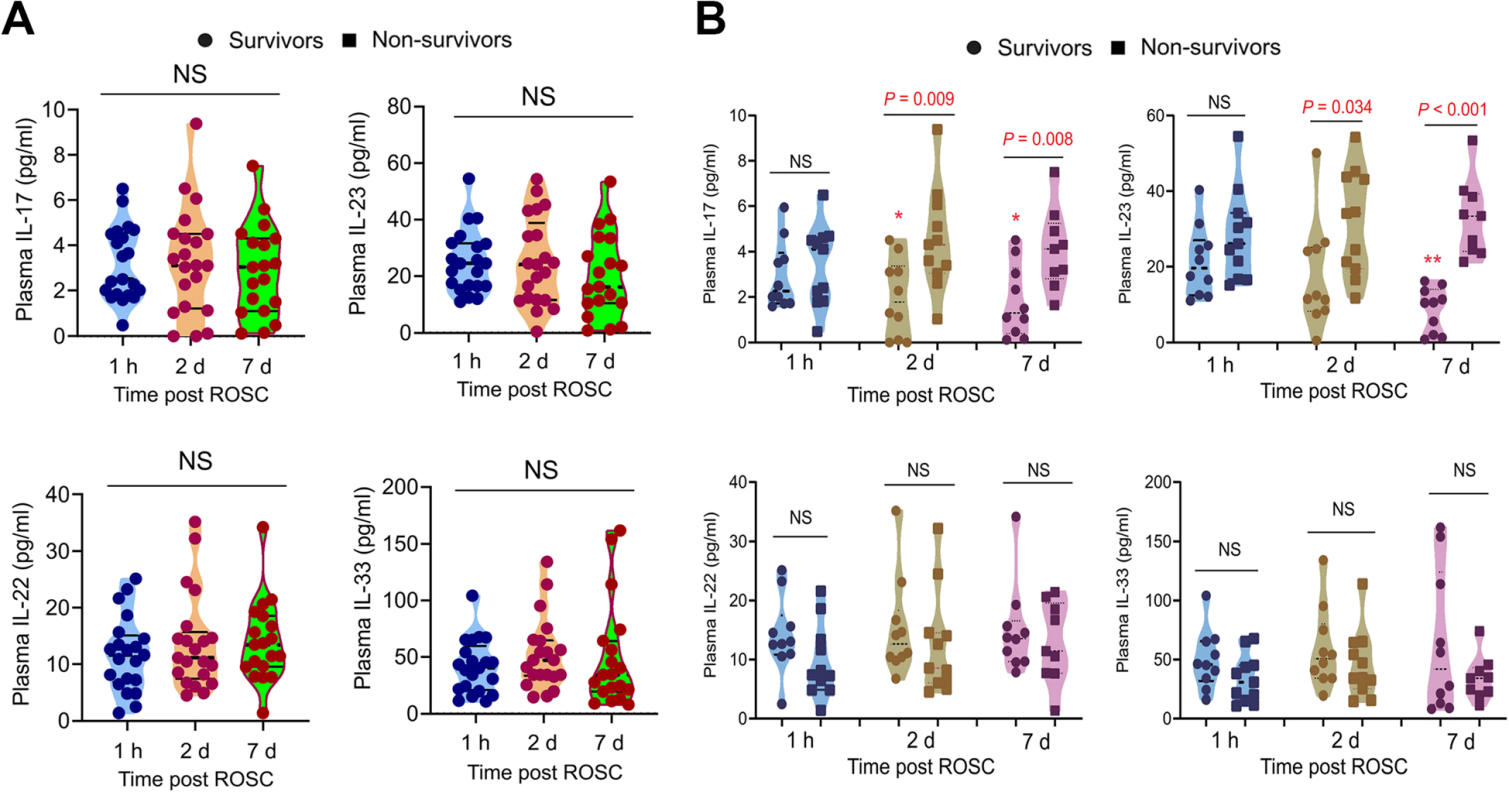
**The plasma levels of four kinds of pro-inflammatory factors in PCAS patients at three time-points.** (**a**) Violin plot shows the plasma levels of IL-17, IL-23, IL-22 and IL-33 in PCAS patients at three time-points (1 h, 2 days and 7 days post ROSC). The thick dotted line in violin plot indicates the median, whereas the two solid thin lines indicate the upper and lower quartiles. (**b**) Scatter plot shows the comparison of plasma levels of IL-17, IL-23, IL-22 and IL-33 in survivors and non-survivors of PCAS patients at three time-points (1 h, 2 days and 7 days post ROSC). **P < 0.01, *P < 0.05 vs 1 h post ROSC. NS, no significance

We monitored the plasma levels of pro-inflammatory factors (including IL-17, IL-22, IL-23 and IL-33) with 1 h, two day and seven day post ROSC respectively in the enrolled 21 PCAS patients. However, the four pro-inflammatory factors plasma concentrations did not differ among the three checked time points (Fig. 1A).

### Post-ROSC plasma levels of IL-17 and IL-23 have different change patterns between survivors and non-survivors

Then, we grouped the 21 PCAS patients into survivors (*n* = 10) and non-survivors (*n* = 11) and analyzed the potential discrepancy between them. The basic features of survivors and non-survivors were illustrated in Table 1. Notably, the plasma IL-17 concentrations in non-survivors at 2 and 7 days post ROSC were significantly higher than those in survivors (Fig. 1B). However, this phenomenon was not observed at 1 h post ROSC (Fig. 1B). Similarly, the plasma IL-23 concentrations in non-survivors were significantly higher than those in survivors at 2 and 7 days post ROSC but not 1 h post ROSC (Fig. 1B). Plasma IL-22 levels post ROSC in both survivors and non-survivors did not change and there was no difference between survivors and non-survivors (Fig. 1B). Also, there was no significant difference in plasma IL-33 levels among 0, 2 and 7 days post ROSC in both survivors and non-survivors and the plasma IL-33 levels did not differ between the survivors and non-survivors (Fig. 1B). There was another interesting phenomenon in plasma IL-17 and IL-23 concentrations that the plasma IL-17 and IL-23 concentrations in survivors declined with time (7 days post ROSC vs 1 h post ROSC, *P* < 0.05) but not in non-survivors (Fig. 1B).

**Table 1:** Basic patient characteristics

	Survivors (n = 10)	Non-survivors (n = 11)	*P*
Age (years)	74.8	70.7	
Sex, m/f	6/4	6/4	
Witnessed by a bystander	9/10	9/11	
Time to ROSC (min)	6.86	24.5	0.0012
APACHE II score	14.14	29.14	< 0.0001
CPC score	2.28	5	0.0012
Out-of-hospital arrest	2/10	4/11	
Ventricular defibrillation	2/10	3/11	
Myocardial infarction	3/10	4/11	
At presentation in ICU			
Cardiogenic shock	2/10	3/11	
Post-resuscitation sepsis	2/10	2/11	
Length of ICU stay, days	10.1	16.2	0.0015
Length of hospital stay, days	19.4	16.3	

Data are expressed as average or absolute number where appropriate. ROSC, return of spontaneous circulation; ICU, intensive care unit; APACHE II, Acute Physiology and Chronic Health Evaluation II; CPC, cerebral performance category

### Renal dysfunction in CA patients post ROSC

**Fig. 2:**
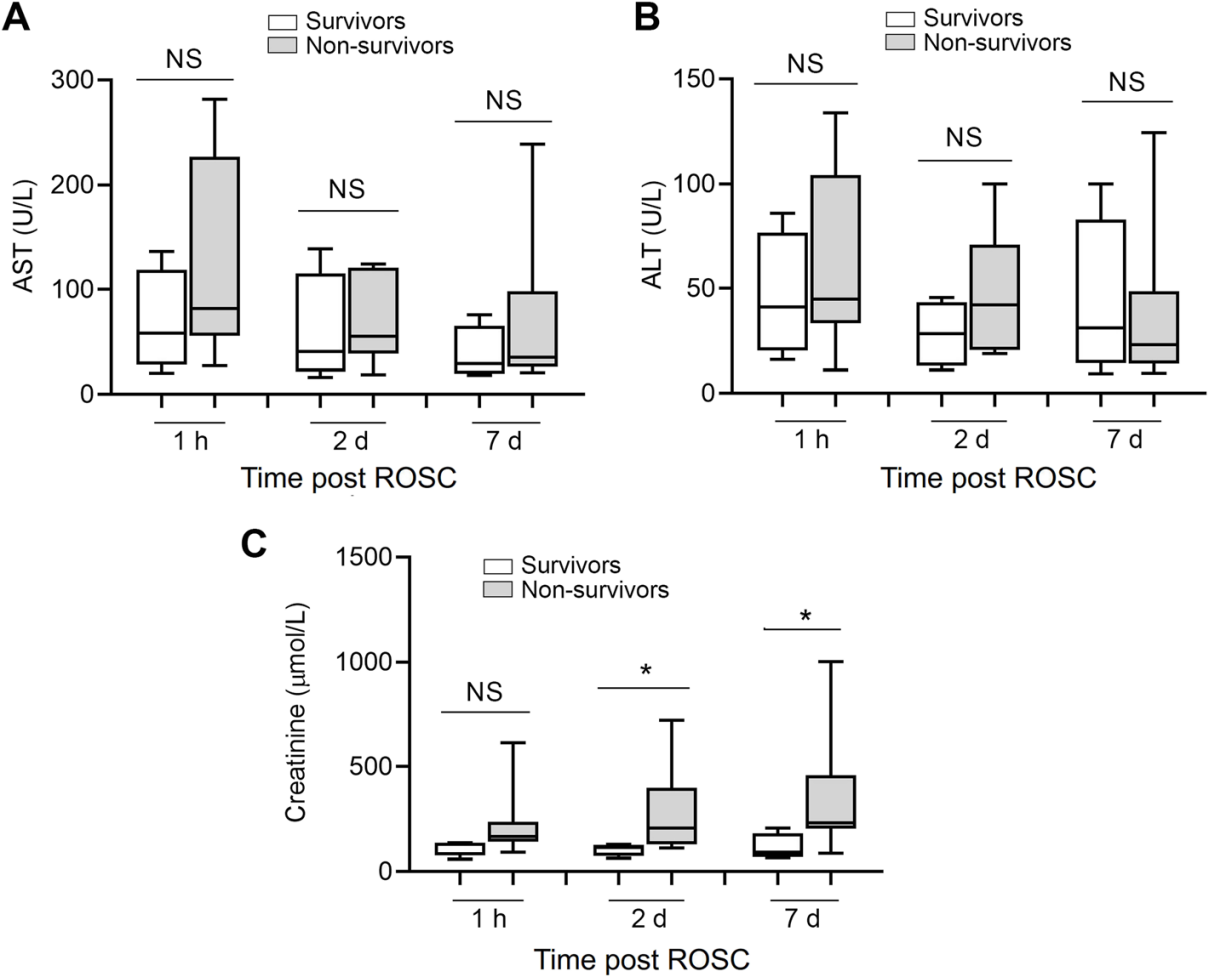
**Comparison of liver or renal dysfunction between survivors and non-survivors at 0, 2 and 7 days post ROSC.** (**a-b**) Plasma concentrations of AST (**a**) and ALT (**b**) survivors and non-survivors. (**c**) Plasma concentrations of creatinine survivors and non-survivors at three time-points. NS, no significance. *P < 0.05 non-survivors vs survivors. NS, no significance

We compared the functions of liver (plasma AST and ALT concentrations) and kidney (plasma creatinine concentration) between survivors and non-survivors. As shown in Fig. 2A-B, the plasma AST and ALT levels did not show significant differences between survivors and non-survivors at 0, 2 and 7 days post ROSC. However, the plasma creatinine levels, an index of renal dysfunction, were increased in non-survivors at 2 and 7 days post ROSC (Fig. 2C). There was no association between plasma creatinine level and IL-23/IL-17 levels (data not shown).

### Post-ROSC plasma IL-17 and IL-23 levels as possible predictors of survival in PCAS patients

**Fig. 3:**
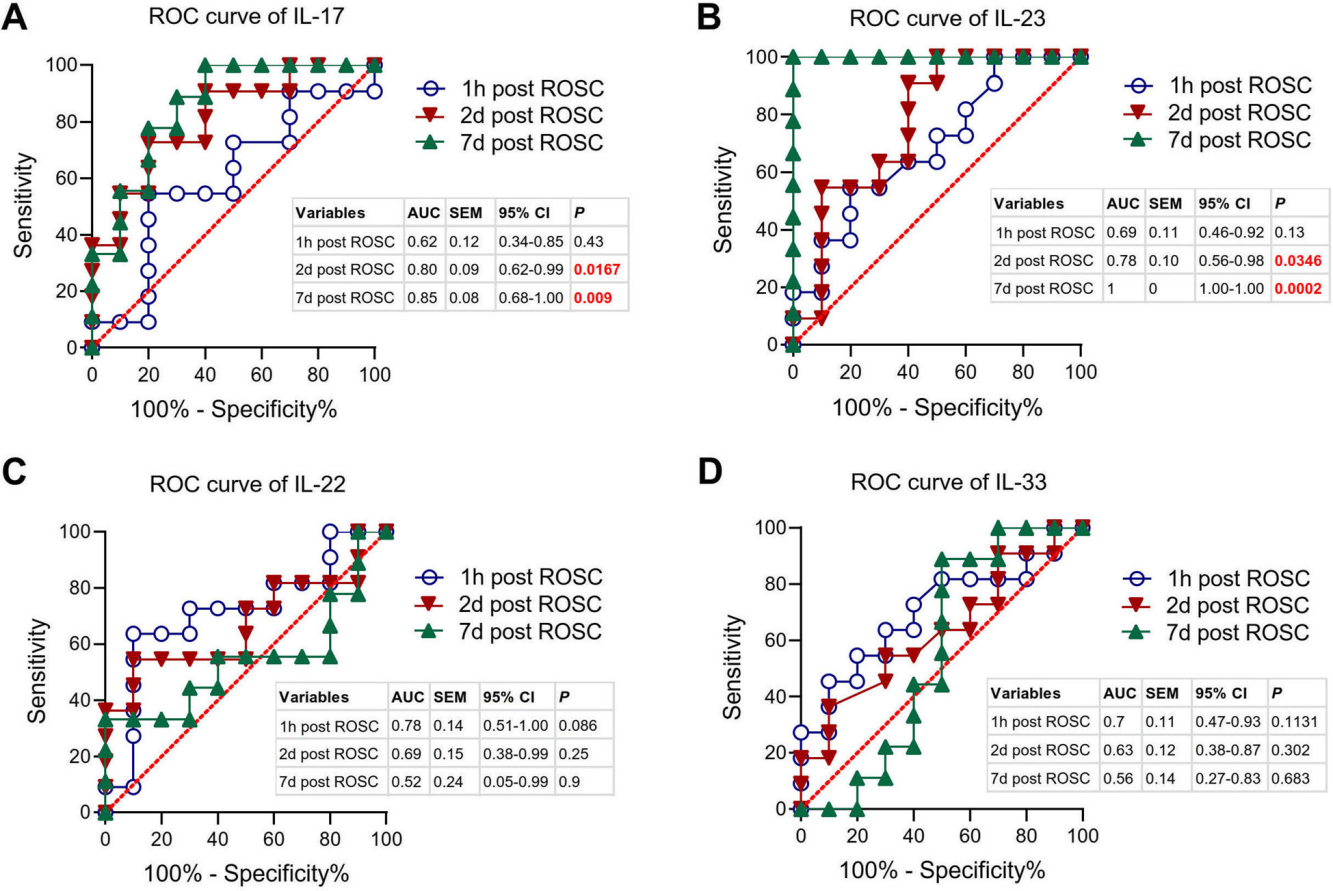
**Plasma levels of IL-17 and IL-23 are potential predictors of survival in PCAS patients.** ROC curves for plasma levels of IL-17, IL-23, IL-22 and IL-33 at three time-points (1 h, 2 days and 7 days post ROSC) were assessed as possible predictors of survival in PCAS patients. The area under the curve (AUC), standard error of the mean (SEM), 95% confidence interval (95% CI) and *P* value were also illustrated

Next, we analyzed whether the plasma levels of IL-17, IL-22, IL-23 and IL-33 might predict mortality. ROC analysis revealed that plasma IL-17 levels at 2 days post ROSC (AUC = 0.8, *P* = 0.0167) and 7 days post ROSC (AUC = 0.85, *P* = 0.009) were good predictors of outcome in PCAS patients (Fig. 3A). Plasma IL-23 levels at 2 days (AUC = 0.78, *P* = 0.0346) or 7 days post ROSC (AUC = 1, *P* = 0.0002) were also good predictors of outcome in PCAS patients (Fig. 3B). Plasma IL-22 and IL-33 levels were unable to predict the outcome of PCAS patients (Fig. 3C-D).

### Post-ROSC plasma IL-17 and IL-23 levels are positively associated with APACHE II score in PCAS patients

**Fig. 4:**
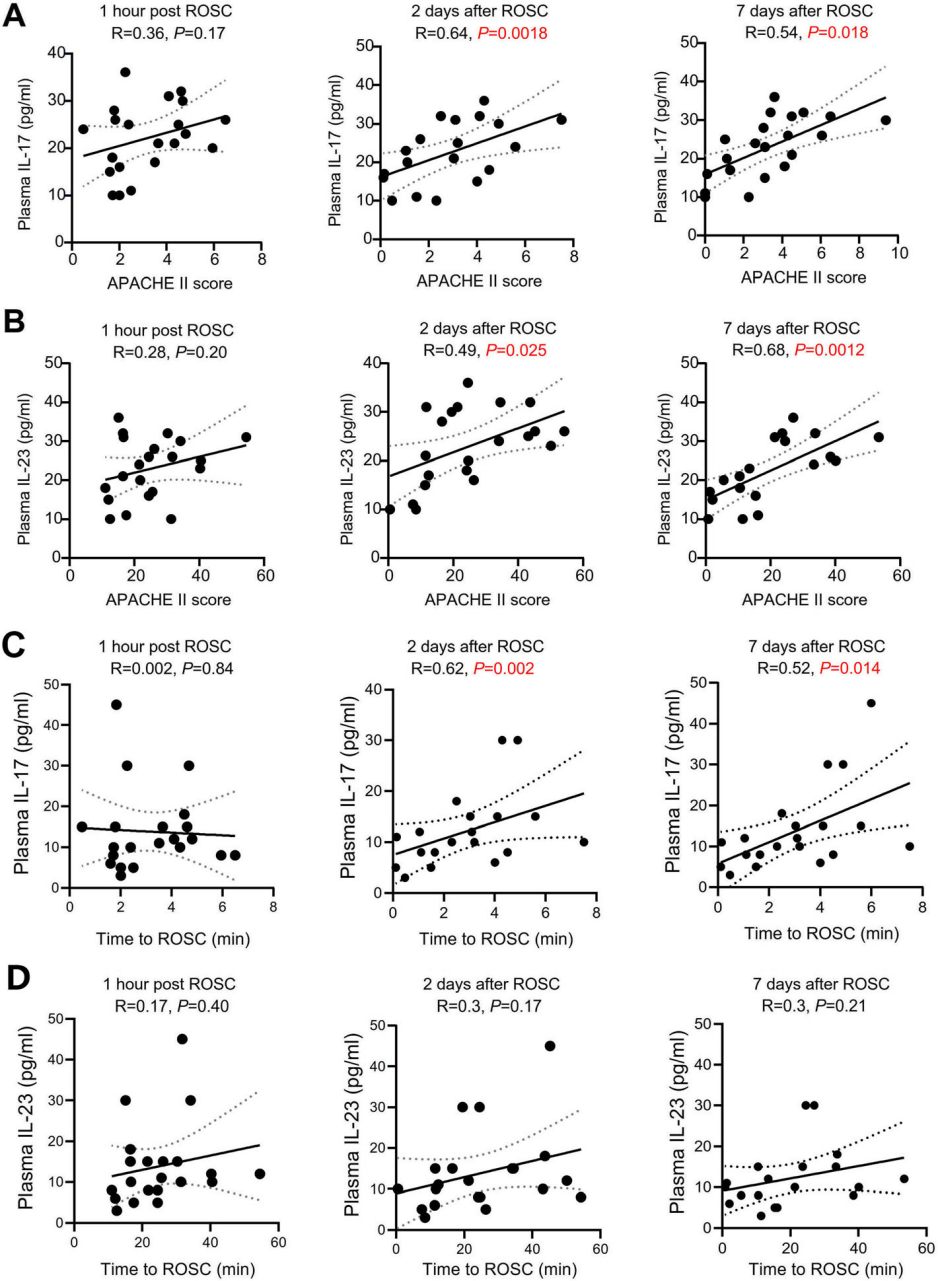
**Association between plasma levels of IL-17/IL-23 axis and APACHE II score or time to ROSC in PCAS patients.** (**a-b**) Pearson linear regression analyses showed there were no associations between plasma IL-17 (**a**) and IL-23 (**b**) levels and APACHE II score at three time-points (1 h, 2 days and 7 days post ROSC). (**c-d**) Pearson linear regression analyses showed there were no associations between plasma IL-17 (**c**) and IL-23 (**d**) levels and time to ROSC at three time-points (1 h, 2 days and 7 days post ROSC)

To study the relationship between post-ROSC plasma IL-17, IL-22, IL-23 and IL-33 levels and mortality, we performed a linear analysis using APACHE II score, a well-known comprehensive index of severity of neurological deficit [26]. In these PCAS patients, the APACHE II score was positively associated with the plasma IL-17 levels at 2 (r2 = 0.75, *P* = 0.0001) or 7 days (r2 = 0.53, *P* = 0.0163) but not 1 h post ROSC (Fig. 4A). A similar association between APACHE II score and plasma IL-23 levels was also observed (Fig. 4B). By contrast, there was no correlation between the plasma levels of IL-22/33 and APACHE II score at the three time-points (Supplemental Fig. 1).

### Post-ROSC plasma IL-17 levels are positively associated with time to ROSC in PCAS patients

Time to ROSC is a strong predictor of neurological outcome after ROSC. We found the time to ROSC correlated with the plasma concentrations of IL-17 levels at 2 and 7 days post ROSC (Fig. 4C). Plasma levels of IL-23, IL-22 and IL-33 did to correlate with the time to ROSC (Fig. 4D and Supplemental Fig. 2).

## Discussion

As a leading cause of death in developed and many developing countries, cardiac diseases are accounted for around 70% of cases of cardiac arrest; coronary artery diseases are the commonest cause. This is the first study to show the elevated plasma IL-17 and IL-23 levels in PCAS patients and that the plasma concentrations of IL-17 and IL-23 were associated with poor clinical outcome following cardiac arrest. Although the plasma IL-17, IL-22, IL-23 and IL-33 levels did not show any changing trend over time in total PCAS patients, we found significant differences of plasma IL-17 and IL-23 levels between survivors and non-survivors patients at 2 and 7 days post ROSC. By contrary, the plasma IL-17 and IL-23 levels at baseline (< 1 h post ROSC) did not differ between survivors and non-survivors.

It is well-established that the ischemia/reperfusion-induced injury in organs such as heart and brain is closely related to activation of neutrophils/macrophages and extensive releases of pro-inflammatory cytokines and inflammasome [27–29]. During this procedure, oxidative stress and endoplasmic reticulum stress also contribute to the cardiac injury [30, 31]. The relationship between inflammation and cardiac arrest has been investigated extensively [32]. Adrie et al. investigated the immune-inflammatory profile in cardiac arrest patients with successfully ROSC and found that high levels of plasma IL-6, IL-8, IL-10, and TNF receptor type II discriminated between survivors and non-survivors [33]. These phenotypes, including the high circulating pro-inflammatory cytokines and the existence of endotoxin in blood, and the absence of ability to product cytokines, mimic the pathophysiology of immunologic and coagulation malfunction observed in sepsis [33]. In agreement with this, TNF-α levels in blood was found to increase during recovery from cardiac arrest in swine model and the increment of TNF-α levels were associated with depression of left ventricle function [34]. Peberdy et al. performed a work to investigate the inflammatory markers including IL-1Ra, IL-6, IL-8, and IL-10 during early stage after ROSC in 102 patients and found baseline IL-6 levels were a good predictor of mortality and strongly associated with in-hospital mortality and poor neurological outcome [35]. Bro-Jeppesen et al. reported baseline plasma IL-6 and IL-10 levels were significantly higher in non-survivors compared with survivors [11, 12]. Melatonin, an anti-oxidant and anti-inflammatory agent, improves neurological outcomes and preserves hippocampal mitochondrial function in cardiac arrest [36], which may be associated with its protection against inflammation [22, 37–39], myocardial ischemia-reperfusion injury [40–43], ventricular fibrillation [44], cardiac hypertrophy [45], dilated cardiomyopathy [46], and mitochondria-related disorders [47, 48]. As neutralization of IL-17 rescues neuroinflammation [49], melatonin may contribute to the complex role of IL-17 signaling in ROSC [50].

In this study, we selected four pro-inflammatory cytokines (IL-17, IL-22, IL-23 and IL-33) to assess whether they can be used to predict the outcome of PCAS patients. We found plasma IL-17 and IL-23 levels of survivors were significantly lower than those of non-survivors. IL-17 is a major pro-inflammatory cytokine secreted by activated T helper 17 (Th17) and contributes to the pathogenesis of both infectious and inflammatory diseases [51]. Meanwhile, IL-23 acts as a maintainer of Th17 cells and facilitates the expansion of Th17 cells [52]. The key roles of IL-23/IL-17 signaling pathway axis has been well-characterized in arthritis, autoimmune skin diseases, inflammatory bowel disease and neuroinflammation [52]. However, there is no information on IL-23/IL-17 signaling pathway axis in cardiac arrest. In our work, we observed that plasma IL-17 levels decreased over time in survivors but increased in non-survivors during the post-cardiac arrest period. Plasma IL-23 levels also decreased over time in survivors at 2 and 7 days post ROSC although we did observe the increasing of plasma IL-23 level in non-survivors. It should be noted that a large number of previous investigations have documented the critical roles of IL-23/IL-17 signaling in ischemic injury. Li et al. found that innate immune component of kidney ischemic injury requires activation of the IL-23/IL-17 signaling pathways for the neutrophil infiltration [53]. IL-23/IL-17 axis aggravated the left ventricular remodeling after myocardial infarction [54]. In brain ischemia condition, IL-23 seemed to function in the immediate stage of ischemia/reperfusion neural injury, whereas IL-17 played a pivotal role in the delayed phase of ischemia/reperfusion injury [55]. Further, neutralization of the IL-17 axis by an IL-17A–blocking antibody diminished the neutrophil recruitment and pro-inflammatory factors release [56]. These results suggest that IL-17/IL-23 axis may play detrimental effects in ischemic injury. Thus, the decreased plasma IL-17 and IL-23 level in survivors implicate a remarkable reduction of organ injury induced by ischemia, whereas the elevation of IL-17 in non-survivors suggests an aggravation of ischemic injury. It should be noted that several other interleukins such as IL-19 and IL-21 also contribute to myocardial injury during ischemia [57–59], so we may need more investigation on them in the future.

Many plasma inflammation markers may predict outcome of patients with cardiac arrest. There were numerous investigations on them. For example, neutrophil lymphocyte ratio, a marker of systemic inflammation, was previously reported to be associated with mortality independently from epinephrine application [60]. Another study showed that neutrophil lymphocyte ratio in survivors was 4.9 (range 0.6–46.5) compared with 8.9 (0.28–96) in non-survivors (*P* = 0.001) [61]. Blood concentrations of C-reactive protein steadily increased in the several days after ROSC in CA patients and there was a much pronounced higher CRP levels in the patients with system infection [62]. In a logistic regression model, high CRP levels on admission were independently associated with poor neurological outcome at 3 months [62]. However, Oppert et al. found that elevations in procalcitonin but not C-reactive protein are associated with pneumonia after cardiopulmonary resuscitation [63]. Consequently, inflammatory factors are likely to be important mediators and predictors for outcome of cardiac arrest patients. Nevertheless, the concentrations of IL-23 and IL-17, which are crucial to the maintenance of the pro-inflammatory feedback loop by maintaining Th17 cells development and homeostasis, have never been studied in patients with cardiac arrest. Our study is the first to investigate the potential relationship of IL-23/17 axis and cardiac arrest outcome.

Considering that cardiopulmonary resuscitation after cardiac arrest may result in a “sepsis-like” syndrome, the critical involvement of IL-17/IL-23 axis in sepsis may help to understand how IL-17/IL-23 axis modulates the tissue injury in PCAS patients. T cells are primed to promote IL-17 production by increasing STAT3-mediated transcriptional regulation in the lungs of mice with sepsis [64]. Supporting this, blocking IL-17 down-regulated inflammation and conferred protection against sepsis and reduced mortality to both neonatal sepsis and endotoxemia by targeting IL-18 [65]. Additionally, shock mice with *Pseudomonas aeruginosa* infection showed early and sustained expression of IL-23 in the spleens, and administration of IL-23-neutralizing antibody protected the mice from Gram-negative endotoxic shock [66]. These evidences suggest that IL-17/IL-23 axis substantially contributes to the immunopathology and mortality of sepsis. As a result, IL-17/IL-23 may functions as an inflammation enhancer and the elevation of them in blood indicates a pro-inflammatory status in PCAS patients. Due to the complex role of IL-17/IL-23 in cardiovascular disorders [67], our findings on IL-17/IL-23 axis in PCAS may bring more information about their features in cardiovascular system.

The catecholamine-induced adrenergic receptor stimulation was a common tool to maintain blood pressure in therapy of cardiac arrest, with catecholamine epinephrine being the commonest drug used in ICU [68]. The role of catecholamine and the changed hemodynamics related to the catecholamine levels in cardiac arrest are important characteristics in resuscitation. As early as in 1989, the elevated plasma catecholamines and resuscitation from prolonged cardiac arrest in dogs was firstly reported by Kern et al. [69]. By contrast, Wu et al. showed that the plasma dopamine levels increased, while plasma epinephrine and norepinephrine levels gradually decreased after recurrent ventricular fibrillation in pigs [70]. This suggests that there is still a dispute in plasma catecholamine in cardiac arrest. The relationship between catecholamines and IL-17/23 axis may be an interesting question which may need further investigation in the future.

The influence of cardiac arrest etiology on the differences in IL-17 and IL-23 levels may be another interesting issue. As we have excluded the patients with severe infection, the elevated blood IL-17 and IL-23 were not derived from bacterial infection or LPS stimulation. In fact, almost all the patients included in our study have cardiovascular-metabolic disorders. The cardiovascular diseases, including atherosclerosis, hypertension, myocardial infarction and even atrial fibrillation, seemed to be tightly associated with the activated IL-17/23 axis [52]. Moreover, metabolic disorders, including obesity and diabetes, were also related with IL-17/23 axis [52]. Since these cardiovascular-metabolic dysfunctions were directly associated with IL-17/23 axis, we considered that the etiology of cardiac arrest may have direct relationship with IL-17 and IL-23 levels. Nevertheless, this question may be a complex issue needing in-depth studies which involves more patients.

In summary, we conclude that plasma IL-17/IL-23 axis is a potential predictor of outcome in PCAS patients. Moreover, targeting IL-17/IL-23 axis may serve as a possible therapeutic target to reduce inflammation during post ROSC period in PCAS patients. In order to test these concepts, additional preclinical experiments and clinical trials with larger cohort sizes will be required.

## Supplementary information

**Additional file 1.** Plasma levels of IL-23 (Fig. 4D) did to correlate with the time to ROSC. Similar results were oberved in plasma levels of IL-22 and IL-33 (Supplemental Fig. 2).

## Data Availability

The datasets used and/or analyzed during the current study are available from the corresponding author on reasonable request.

## Abbreviations

APACHE: Acute physiology and chronic health evaluation
AUC: Area under the curve
CPR: Cardiopulmonary resuscitation
IFN: Interferon
IL: Interleukin
PCAS: Post-cardiac arrest syndrome
ROC: Receiver operating characteristic
ROSC: Return of spontaneous circulation
TNF-α: Tumor necrosis factor-α
