# Supplementary material for: High plasma levels of pro-inflammatory factors interleukin-17 and interleukin-23 are associated with poor outcome of cardiac-arrest patients: a single center experience
Source: BMC Cardiovasc Disord. 2020 Apr 15;20:170. doi: 10.1186/s12872-020-01451-y (PMC7158084; doi:10.1186/s12872-020-01451-y)
Supplement: Supplementary file 1 — Additional file 1. Plasma levels of IL-23 (Fig. 4D) did to correlate with the time to ROSC. Similar results were oberved in plasma levels of IL-22 and IL-33 (Supplemental Fig. 2). [file 12872_2020_1451_MOESM1_ESM.docx]

**Supplemental data**

**High plasma levels of pro-inflammatory factors interleukin-17 and interleukin-23 are associated with poor outcome of cardiac-arrest patients: a single center experience**

Yu-Gang Zhuang,^1^ Yuan-Zhuo Chen,^1^ Shu-Qin Zhou,^1^ Hu Peng,^1^ Yan-Qing Chen,^1^ Dong-Jie Li^1,2,3^

*^1^Department of Emergency Medicine, Shanghai Tenth People's Hospital, School of Medicine, Tongji University, Shanghai, China*

*^2^Key Laboratory of Molecular Pharmacology and Drug Evaluation (Yantai University), Ministry of Education, Yantai University, Yantai, China*

*^3^Department of Pharmacy, Shanghai Tenth People's Hospital, School of Medicine, Tongji University, Shanghai, China*

**Corresponding author:**

Prof. Dong-Jie Li, Ph.D, MD.

**Affiliation**: Department of Pharmacy, Shanghai Tenth People's Hospital, School of Medicine, Tongji University, Shanghai, China

**Address:** 301 Yanchangzhong Road, Jingan District, Shanghai, China

**Tel:** 86-21-66302570

**Email:** [djli@tongji.edu.cn](mailto:djli@tongji.edu.cn)

**Supplemental Figure 1**


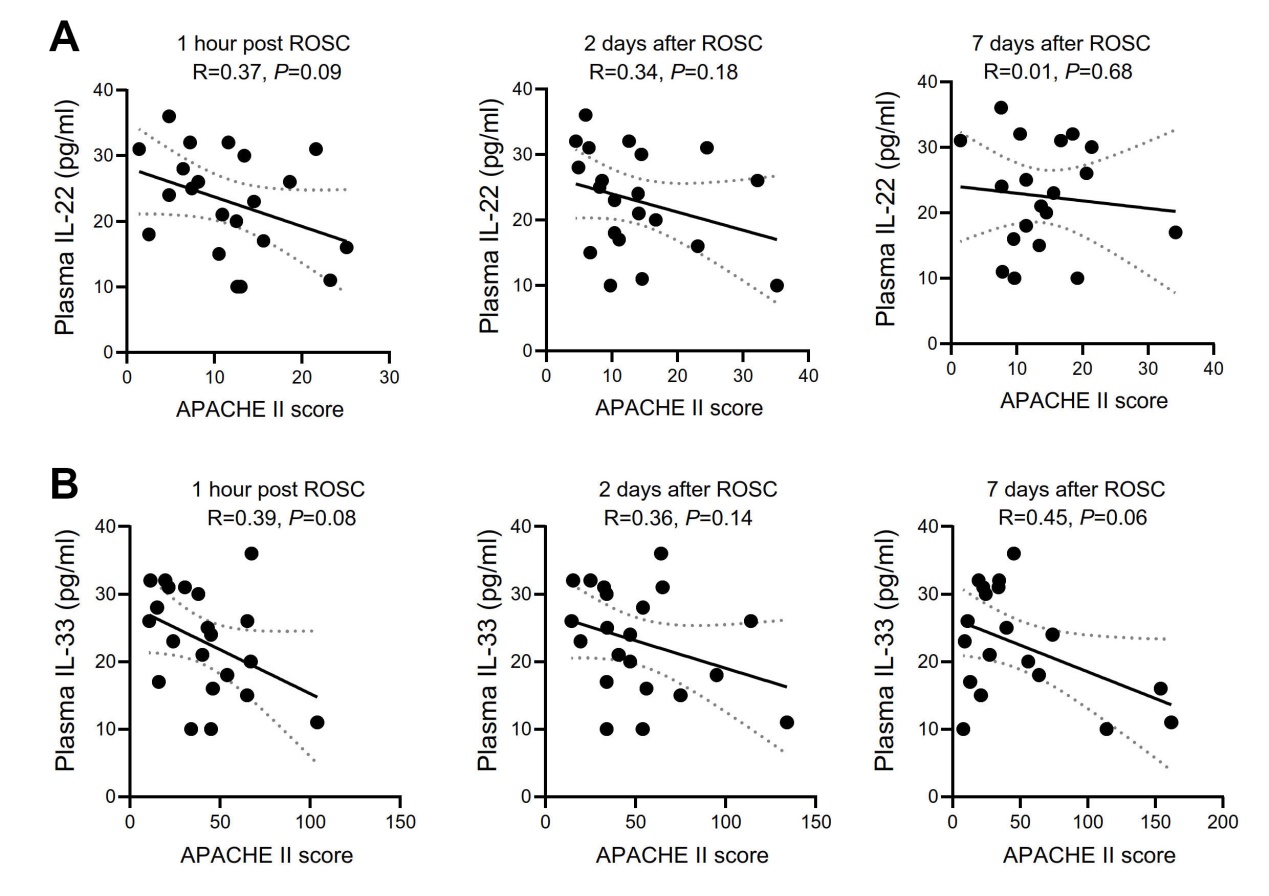


(A) No significant association between plasma IL-22 levels and APACHE II score.

(B) No significant association between plasma IL-33 levels and APACHE II score.

**Supplemental Figure 2**


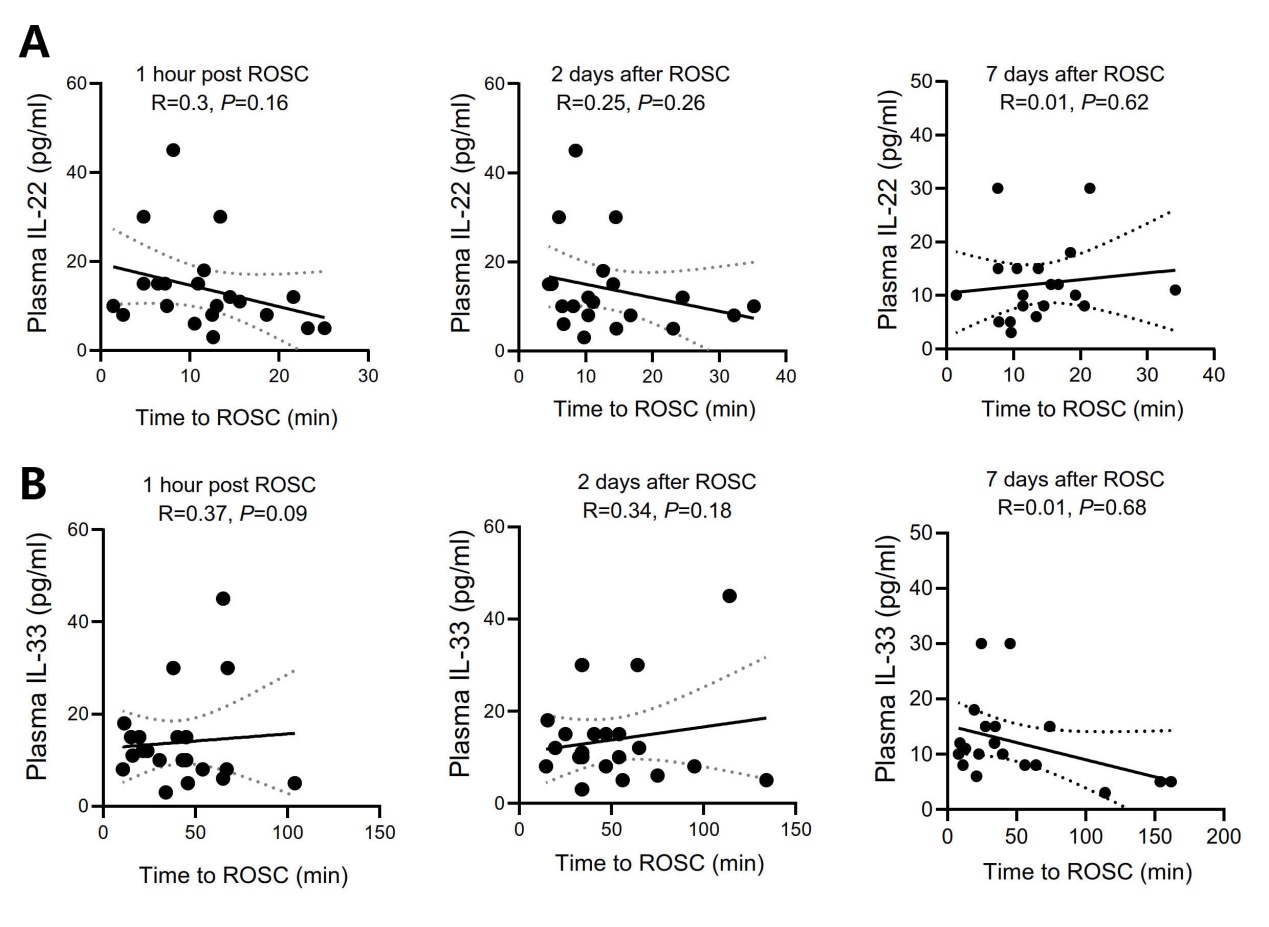


(A) No significant association between plasma IL-22 levels and time to ROSC.

(B) No significant association between plasma IL-33 levels and time to ROSC.
